# Effect of Whitlockite as a new bone substitute for bone formation in spinal fusion and ectopic ossification animal model

**DOI:** 10.1186/s40824-021-00237-3

**Published:** 2021-10-21

**Authors:** Yuan-Zhe Jin, Guang-Bin Zheng, Minjoon Cho, Jae Hyup Lee

**Affiliations:** 1 Department of Orthopedic Surgery, College of Medicine, Seoul National University, Seoul, 03080 South Korea; 2 The First Hospital of Jilin University, Changchun City, 130021 China; 3 Department of Orthopaedics, Taizhou Hospial of Zhejiang Province, Linhai, Zhejiang, 317000 China; 4 Department of Orthopedic Surgery, SMG-SNU Boramae Medical Center, Boramae-ro 5-gil 20, Dongjak-gu, Seoul, 07061 South Korea

**Keywords:** Bone, Calcium phosphate, Hydroxyapatite, Whitlockite

## Abstract

**Background:**

Bone substrates like hydroxyapatite and tricalcium phosphate have been widely used for promoting spinal fusion and reducing the complications caused by autograft. Whitlockite has been reported to promote better bone formation in rat calvaria models compare with them, but no study investigated its effect on spinal fusion yet. Also, the higher osteoinductivity of whitlockite raised concern of ectopic ossification, which was a complication of spinal fusion surgery that should be avoided.

**Methods:**

In this study, we compared the osteoinductivity of whitlockite, hydroxyapatite, and tricalcium phosphate porous particles with SD rat spine posterolateral fusion model and investigated whether whitlockite could induce ectopic ossification with SD rat abdominal pouch model.

**Results:**

The micro-CT result from the posterolateral fusion model showed whitlockite had slightly but significantly higher percent bone volume than tricalcium phosphate, though none of the materials formed successful fusion with surrounding bone tissue. The histology results showed the bone formed on the cortical surface of the transverse process but did not form a bridge between the processes. The result from the abdominal pouch model showed whitlockite did not induce ectopic bone formation.

**Conclusion:**

Whitlockite had a potential of being a better bone substrate hydroxyapatite and tricalcium phosphate in spinal fusion with low risk of inducing ectopic ossification.

## Background

Spine fusion has been widely used to treat trauma, infection, tumour, deformity, and degenerative diseases [1]. However, failure in bone fusion causes pseudarthrosis, which causes pain, instability, and disability [2]. The incident ratio of pseudarthrosis was reported as 17% in adult deformity correction [3], and 5 to 34% in degenerative indications [1]. Spinal fusion is different from bone fracture, the fusion procedure requires bone grafting to add osteogenic potential [1]. Osteoinductive growth factors like bone morphometric protein 2 have been widely used in the clinic, but it comes with the risk of complications such as retrograde ejaculation or heterotopic ossification [4]. Stem cell therapy also showed potential in clinical applications but it was limited by the complications caused by cell harvesting, in vitro expansion procedures, and donor-related heterogeneity [5]. Therefore, autograft was deemed as the “gold standard” for fusion and was used most widely in the clinic due to its reliable effect [6–8]. However, the autograft lacks in amount and could cause donor site complications, like pain, bone loss, hematomas, infection, fracture, neurovascular injury [6]. Additionally, in the case that using the bone from the anterior superior iliac spine the autograft might lack mechanical strength because there is no solid bony structure [6]. Other bone grafts include allograft and xenograft. Allograft was the bone-derived from humans and was a suitable alternative to autograft, but allograft has high a cost and a risk of disease transmission. Xenograft is easily available, and osteoconductive, has good mechanical properties and low cost, but the effect of the xenograft is contradicted and has the risk of zoonose diseases transmitted to human, it was rarely used in clinic [6]. Therefore, ceramic bone substitute including hydroxyapatite (HA), tricalcium phosphate (TCP), calcium pyrophosphate, and bioglass ceramics has been used for promoting spinal fusion [9–13]. The HA was the primary mineral component of teeth and bone, due to its relatively high Ca/P ratio, its resorption rate was low and it was reported that only 5.4% of HA implants was reduced in rabbit cancellous bone in 6 months [14]. The TCP was degradable, but it lacks mechanical strength, and therefore, neither of them was optimal material. Whitlockite (WH) was the second abundant mineral component in human bone occupies approximately 25% of human bone, but its effect was less investigated due to its difficulty in synthesis [15]. But recently, Jang et al. discovered WH could be synthesized with the acidic aqueous system with an excessive amount of Mg^2+^ ion exist, and subsequent studies showed WH induced better bone regeneration than HAP and TCP in rat calvaria defect model [16, 17]. Since WH has been shown to have positive effects on the enhancement of bone formation in a rat calvaria model, it may represent a potential bone substitute material to be used for the spinal fusion. However, as far as we know, no study investigated the effect of WH in a spinal fusion animal model. Since the tissue regeneration capacity of bone substitutes might be different in different fracture models, the effect of WH on spinal fusion should be investigated in a proper spine surgery model [18]. Additionally, in a previous study, the authors even raised the concern of ectopic ossification induced by WH [17]. Ectopic ossification should be avoided in spine surgery because it can decrease the range of motion and even induce neurologic symptoms if the ossification outgrows into the spinal canal [19]. Therefore, in this study we investigated whether WH could maintain its advance in rat spine posterolateral fusion model and whether the WH could induce ectopic ossification in rat muscle model.

## Methods

### Materials

Fabrication of the bone substitutes was identical to a previous study [16]. The porous implants for animal experiments were synthesized WH nanoparticles, commercially purchased hydroxyapatite (HAP: Ca10(PO4)6(OH)2, Sigma-Aldrich) and β-tricalcium phosphate (β-TCP: Ca3(PO4)2, Sigma-Aldrich). Each ceramic powder was mixed with polymethyl methacrylate (PMMA, Bead & Micro, 330 μm diameter) a weight ratio of 1:2. The PMMA-ceramic mixture was fabricated into a cylindrical pellet by applying a pressure of 2 tons for 3 s in a cylindrical mold. The PMMA beads were burned out with a sintering process up to 700 °C, and then the porous in each ceramic were formed. The porous scaffolds were crushed and the 1 mm size particles were chosen with two sieves with 1.18 mm pore size and 0.89 mm pore size.

### Rat abdominal pouch model

**Fig. 1:**
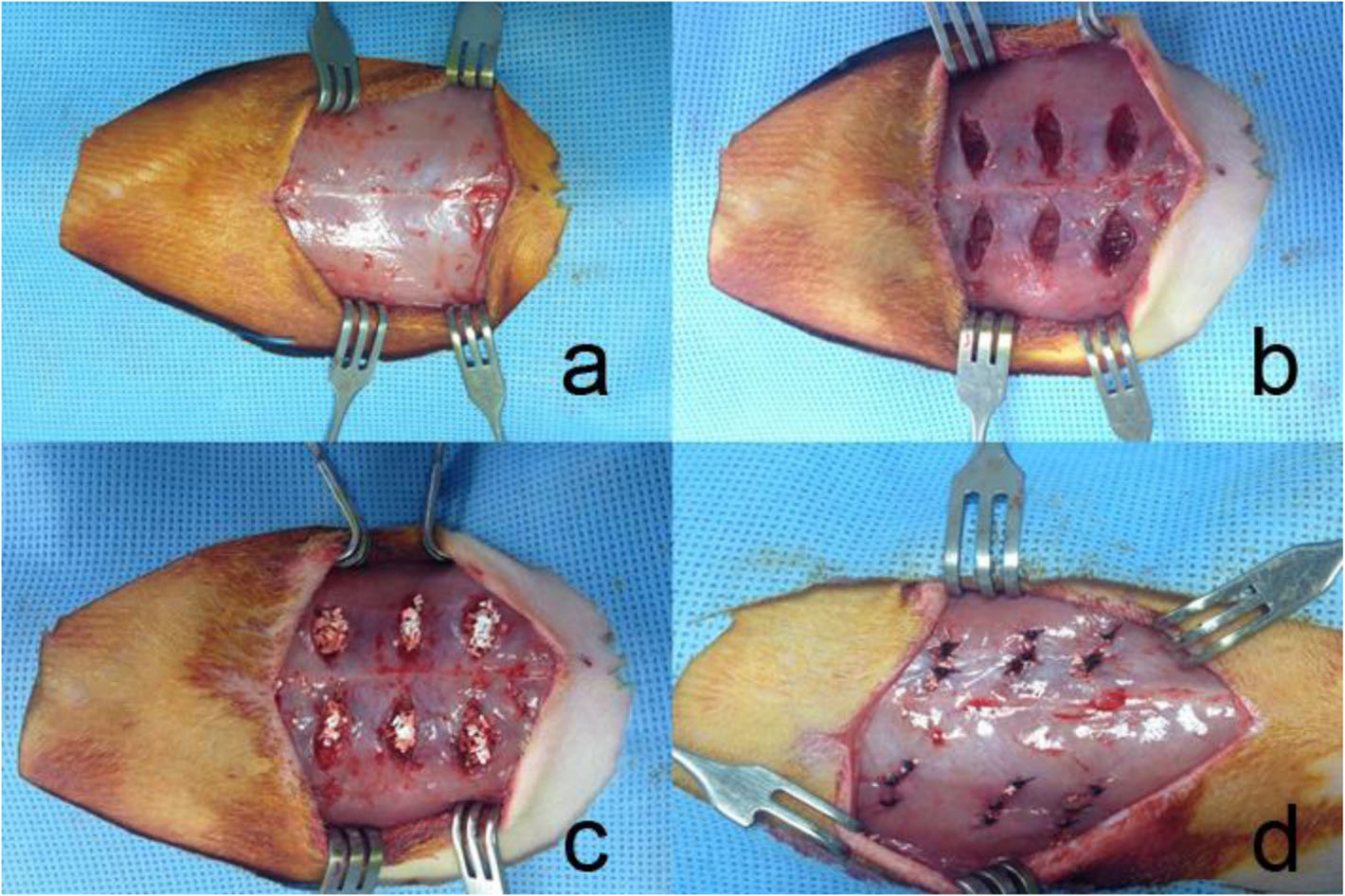
Experiment images of abdominal pouch model. **a** the abdominal skin was opened without interrupting muscle tissue. **b** six pouches in the muscle tissue was made without hurting peritoneum. **c** 15 mg bone substitute was implanted in the muscle pouch. d, the muscle was sutured, and the defect was closed layer by layer

Procedures in the rat abdominal pouch model were approved by the international animal care and use committee (SNUH IACUC No.13–0193). Totally 24 rats were used in this experiment and were equally assigned in the three groups (HA, TCP, WH). Seven weeks old male SD rats were purchased and were kept in a 12:12 light/dark cycle, specific pathogen-free (SPF) room and were provided abundant food and water. The rats were used in their 8th week. After anesthetized with zoletil-xylazine (20 mg/kg Zoletil and 10 mg/kg xylazine) mixture solution, the fur on the abdomen was shaved. After opening the skin, 6 intramuscular pouches were made in the external abdominal oblique. 15 mg of each implant was implanted in the pouch. (Fig. 1) Then the muscle and the skin were sutured layer by layer and cefazolin (100 mg/kg) was injected. The rats were kept under the same condition and were sacrificed 60 days after the experiment.

### Rat posterolateral spine fusion model

**Fig. 2:**
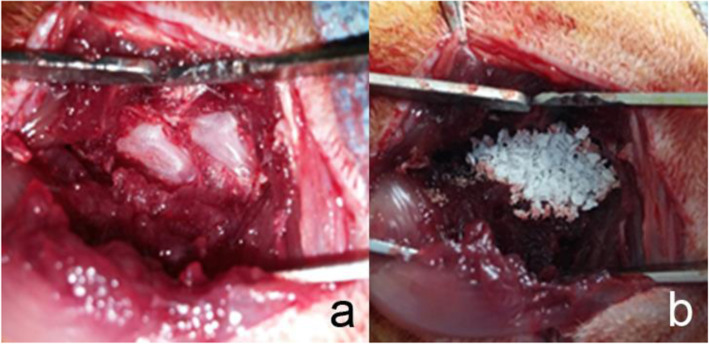
Experiment images of PLF model. **a** L4-L5 level was exposed and the transverse processes were decorticated. **b** 50 mg of bone substitutes was implanted in the decorticated site and the soft tissue was closed layer by layer

The procedure in this rat PLF model was approved by the international animal care and use committee (SNUH IACUC 15–0115-S1A0). Totally 55 rats were used in this experiment, The rats were raised in the same condition as the rat abdominal pouch model. Eight weeks old male SD rats were anesthetized with zoletil-xylazine (20 mg/kg Zoletil and 10 mg/kg xylazine) mixture solution, the fur on the back was shaved. A vertical incision was applied on the skin 2–3 cm above the iliac crest level, and the iliocostal muscle was separated to expose the transverse process. After decorticating the transverse process on both sides of L4 and L5, 50 mg of each implant was implanted in each side. (Fig. 2) After the muscle and skin were sutured layer by layer, the cefazoline (100 mg/kg) was injected. The rats were sacrificed 8 weeks after the experiment.

### Micro-CT

The samples in the rat abdominal pouch model were scanned with Skyscan 1172 (Bruker) at a setting of 9.90 μm pixel size, 59 kV voltage, and 167 μA current. The region of interest (ROI) was set as a rectangular with 11.01 mm width and 8.595 length. The volume of newly formed bone on the surface of each implant was measured.

The samples from rat posterolateral spinal fusion model with the same micro-CT scanner, and were scanned at a setting of 11.55 μm pixel size, 70 kV voltage, and 167 μA current. The ROI was set as a rectangular with 2 mm width and 3 mm length. The newly formed bone in the middle of two transverse processes was analyzed with parameters of percent bone volume (BV/TV), trabecular number (Tb.N), trabecular thickness (Tb.Th), and trabecular separation (Tb.Sp).

### Histology

The abdominal samples were fixed with 10% formalin solution and were decalcified with Calci-Clear Rapid solution (national diagnostics, USA) for 1 week. Then the samples were dehydrated sequentially from 80 to 100% ethyl alcohol and were cleared with xylene and were embedded with paraffin. Then the samples were sectioned with a microtome (Leica RM2255, Leica Biosystems, USA) at a thickness of 4 μm, and were stained with haematoxylin and eosin.

The lumbar spine samples were fixed with 10% formalin and were sequentially dehydrated from 80 to 100% ethyl alcohol. Then the samples were infiltrated and embedded in Technovit 7200 resin (EXAKT, Germany), and the resin was solidified with a polymerization system (EXAKT, Germany). After solidification, the resins were sectioned to 200 μm thick slices with a cutting system (EXAKT, Germany), and the slides were ground to 50 μm thick with a grinding system ((EXAKT, Germany). Then the slices were stained with hematoxylin and eosin.

### Statistic

In this study, we used ANOVA to compare between three groups, with Bonferroni post hoc analysis. (SPSS, IBM) *P*-value less than 0.05 was deemed as statistically significant.

## Results

### Rat PLF model

**Fig. 3:**
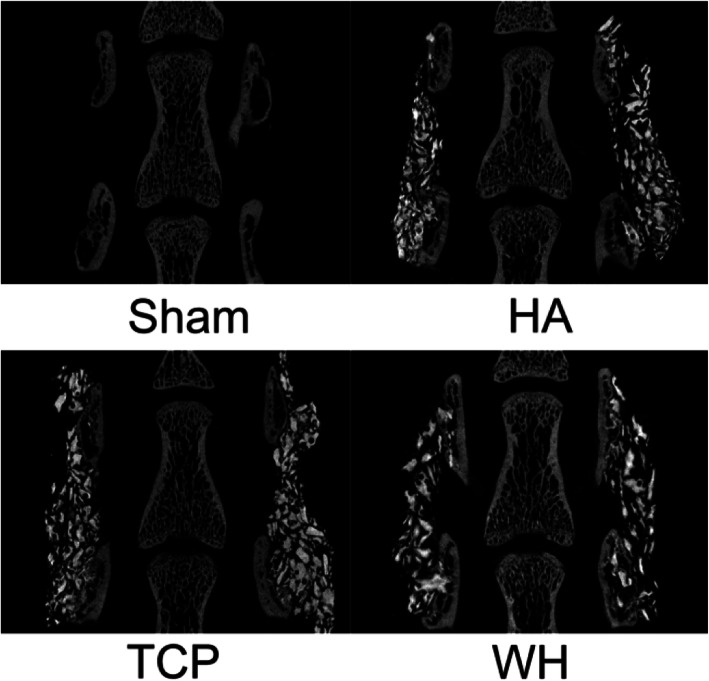
Micro-CT images of rat PLF model. No bone was formed in sham group. All three groups with implant showed bone formed on the surface of decorticated surface and encompassed some implant particles. No significant difference could be observed between the groups. No group formed bridge between the transverse processes

The WH group had the highest percent bone volume (BV/TV) and trabecular bone thickness (Tb.Th) among the groups and they were all significantly higher and narrower than those in the TCP group, respectively. Almost no bone was formed in the sham group and therefore, the difference of parameters between the sham group and the other groups were all statistically significant. (Table 1) However, the bone formed was not enough to bridge between the two transverse processes in any groups, and therefore, no fusion was achieved with any of the implants (Fig. 3).

**Table 1:** Micro-CT result of PLF model

Group (No.)	Mean (SD)			
	BV/TV (%)	Tb.Th	Tb.Sp	Tb.N
HA (26)	6.536(3.453)	0.029 (0.002)	0.372 (0.173)	2.172 (1.084)
TCP (31)	5.992 (3.767)	0.03 (0.002)	0.437 (0.191)	1.981 (1.17)
WH (32)	8.517 (3.484)^#^	0.031 (0.003)	0.319 (0.151)^#^	2.716 (1.002)^#^
SHAM (20)	0.028 (0.108)^$^	0.005 (0.01)^$^	0.863 (0.045)^$^	0.01 (0.039)^$^

All parameters of sham group were significantly different from those of other groups

#: *p* < 0.05, compare with TCP group

^$^: *p* < 0.05, compare with all other groups

**Fig. 4:**
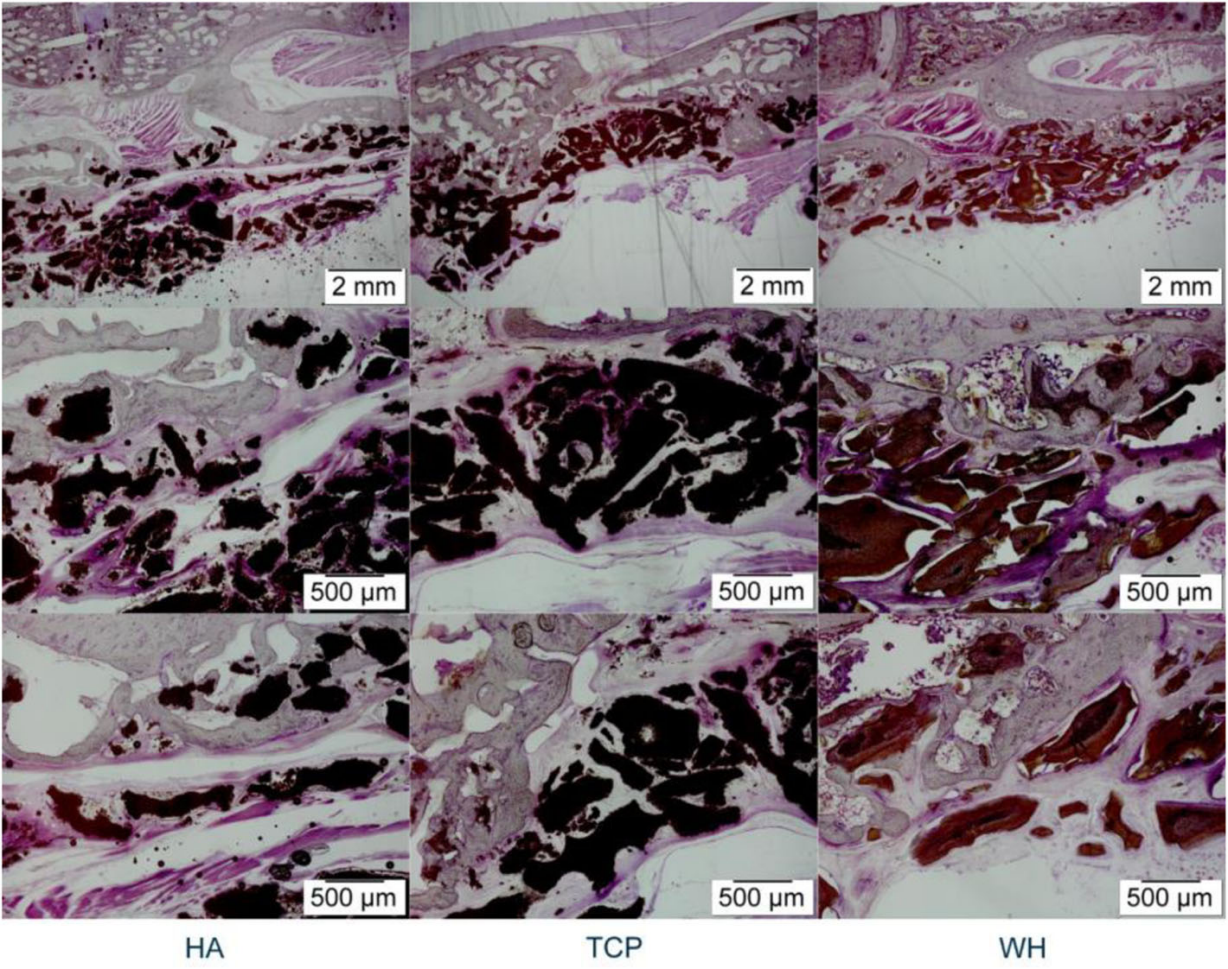
Image of undecalcified H&E staining histology section. All three groups showed bone formation on the decorticated surface. The implant particles were encompassed by the newly formed bone. None of the group showed formation of bridge between the transverse processes

As can be seen in histology sections, the bone in HA, TCP, and WH groups all formed just on the decorticated surface of transverse processes, which was similar to the micro-CT result (Fig. 4). Combine the results from micro-CT and histology sections, though the WH group might form statistically significant more bone than the TCP group, the difference was minimal and none of the groups achieved fusion in the target fusion site.

### Rat abdominal muscle pouch model

**Fig. 5:**
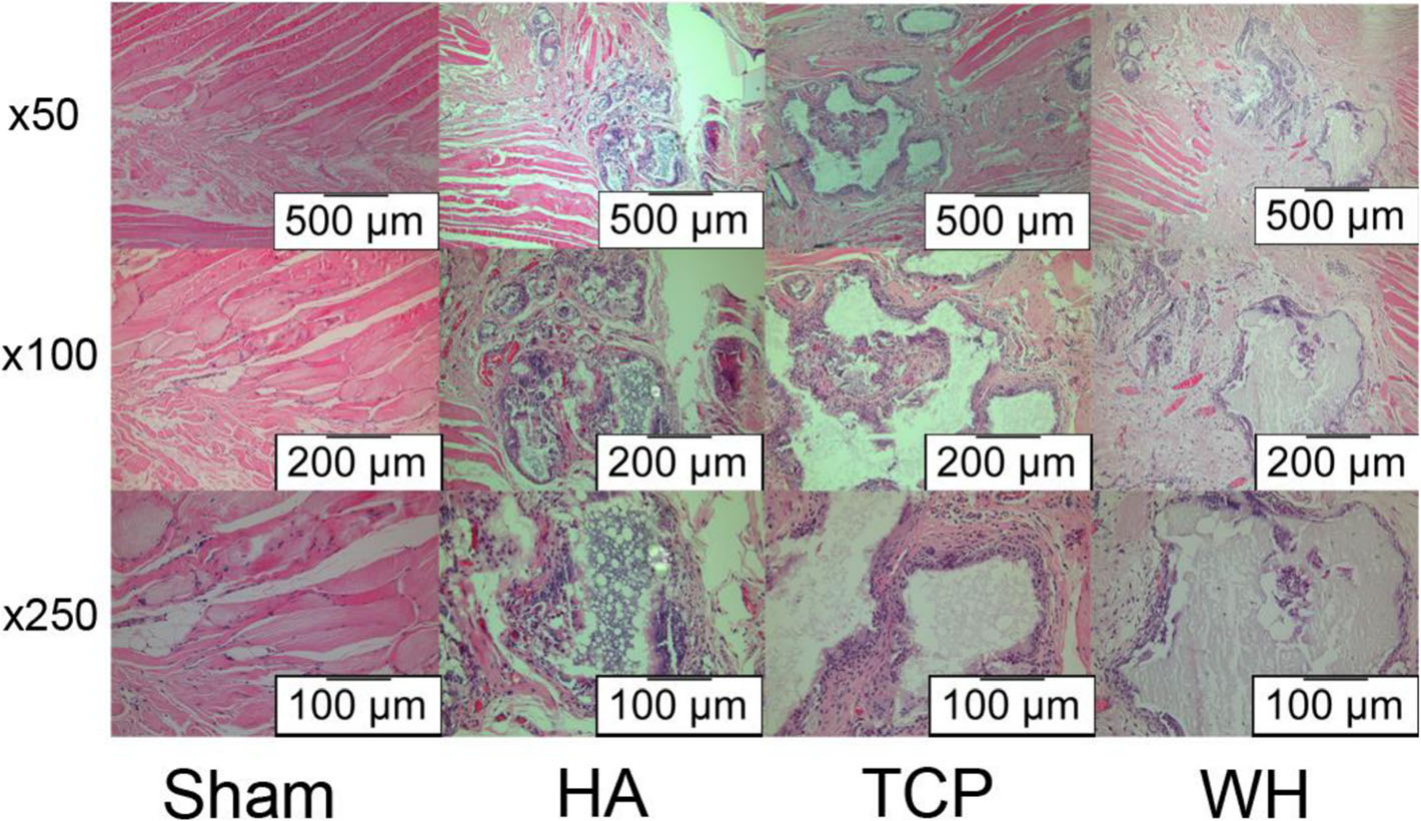
Images of decalcified H&E staining histology section. No bone formation could be observed around the implanted material

Bone was barely formed around the implants in any group. In the micro-CT result, BV/TV was not exceeded 0.5% in any group. The BV/TV in TCP was the highest and was significantly higher than HA (*p* < 0.05) and BV/TV in the WH group was secondly high among the groups. (Table 2) In the histology figure, the implants were decomposed by the decalcification process with a hollow cavity remained. Around the implanted sites, abundant eosinophile granulocyte-like cells was observed around the implants with no obvious difference between the groups, and no bone tissue was observed (Fig. 5).

**Table 2:** Micro-CT result of rat abdominal muscle model

Group (No.)	BV/TV(%)
	Mean (SD)
HA (16)	0.33 (0.11)
TCP (16)	0.46 (0.11)*
WH (16)	0.4 (0.1)

* *p* < 0.05, compare with HA group

## Discussion

WH has been reported to have better osteoinductivity than HA and TCP and therefore, might have the potential of being a new bone substitute material. However, the effect of WH was only examined with the calvaria defect model before and it should be investigated in other animal models for better understanding [16, 17, 20]. Additionally, due to the superior osteoinductivity of WH, it also raised concern about ectopic ossification [17]. Therefore, we compared the osteoinductivity of WH with that of HA and TCP with the rat PLF model and evaluated whether WH could induce ectopic ossification in the abdominal pouch model.

The micro-CT result in the PLF model showed WH had slightly but significantly higher BV/TV than TCP and had insignificantly higher bone formation than HA. It should be noted that though the WH showed significantly higher BV/TV than the TCP group, the difference was not in big magnitude, and therefore, it was too soon to say the WH had superior osteoinductivity than TCP in the rat PLF model. Also, the TCP was reported to degrade faster than HA and WH, which might be another reason that WH had higher BV/TV than TCP [16].

The histology sections showed the bone formed on the decorticated surface but did not form a bridge between the two transverse processes. The unsuccessful fusion might be related to the shape of the implant. Because in the previous study, block-shaped HA were implanted in rabbits and showed successful fusion in 6 weeks [21]. The possible reason that particle-shaped bone substitutes had lower osteoconductivity than block-shaped ones, might be the movement of the particles with the motion of animals. Therefore, it indicated the importance of choosing the proper shape of implant for the best effect of the bone substitute.

The micro-CT result from the abdominal pouch model showed that bone barely formed in the muscle tissue. In the histology sections, no bone tissue could be observed. Due to the superior osteoinductivity of WH than HA, a previous study highlighted the importance of evaluating the risk of WH inducing ectopic ossification [17]. Our results indicated the WH might not be able to induce ectopic ossification, and therefore, might be as biocompatible as HA and TCP might be safe to use in treating bone defect.

Our findings proved the stimulating effect of WH on bone regeneration in a PLF animal model without no obvious inflammatory reaction was observed in the abdominal model. It could be related with the elevated level of the ions of Mg^2+^ and PO_4_^3−^ released by WH and the phase transformation of it [17, 22]. Additionally, the released Mg^2+^ and PO_4_^3−^ also inhibited osteoclastic differentiation [22].

## Conclusion

This study compared the effect of WH with HA and TCP with rat PLF model and ectopic ossification model, as far as we know, was the first study investigated the effect of WH with two animal models other than rat calvaria defect model. The result indicated WH provided comparable osteoinductivity with HA and TCP rat PLF model and possessed a low risk of ectopic ossification. Therefore, WH could be a suitable bone substitute material as TCP and HA.

## Data Availability

The datasets used and/or analysed during the current study areavailable from the corresponding author on reasonable request.

## Abbreviations

HA: Hydroxyapatite
TCP: Tricalcium phosphate
PLF: Posterolateral fusion
PMMA: Polymethyl methacrylate
ROI: Region of interest
SPF: Specific pathogen free
WH: Whitlockite
